# Expression of the ISPpu9 transposase of *Pseudomonas putida* KT2440 is regulated by two small RNAs and the secondary structure of the mRNA 5′-untranslated region

**DOI:** 10.1093/nar/gkab672

**Published:** 2021-08-11

**Authors:** Guillermo Gómez-García, Angel Ruiz-Enamorado, Luis Yuste, Fernando Rojo, Renata Moreno

**Affiliations:** Department of Microbial Biotechnology, Centro Nacional de Biotecnología, CSIC, Madrid 28049, Spain; Department of Microbial Biotechnology, Centro Nacional de Biotecnología, CSIC, Madrid 28049, Spain; Department of Microbial Biotechnology, Centro Nacional de Biotecnología, CSIC, Madrid 28049, Spain; Department of Microbial Biotechnology, Centro Nacional de Biotecnología, CSIC, Madrid 28049, Spain; Department of Microbial Biotechnology, Centro Nacional de Biotecnología, CSIC, Madrid 28049, Spain

## Abstract

Insertion sequences (ISs) are mobile genetic elements that only carry the information required for their own transposition. *Pseudomonas putida* KT2440, a model bacterium, has seven copies of an IS called ISPpu9 inserted into repetitive extragenic palindromic sequences. This work shows that the gene for ISPpu9 transposase, *tnp*, is regulated by two small RNAs (sRNAs) named Asr9 and Ssr9, which are encoded upstream and downstream of *tnp*, respectively. The *tnp* mRNA has a long 5′-untranslated region (5′-UTR) that can fold into a secondary structure that likely includes the ribosome-binding site (RBS). Mutations weakening this structure increased *tnp* mRNA translation. Asr9, an antisense sRNA complementary to the 5′-UTR, was shown to be very stable. Eliminating Asr9 considerably reduced *tnp* mRNA translation, suggesting that it helps to unfold this secondary structure, exposing the RBS. Ectopic overproduction of Asr9 increased the transposition frequency of a new ISPpu9 entering the cell by conjugation, suggesting improved *tnp* expression. Ssr9 has significant complementarity to Asr9 and annealed to it *in vitro* forming an RNA duplex; this would sequester it and possibly facilitate its degradation. Thus, the antisense Asr9 sRNA likely facilitates *tnp* expression, improving transposition, while Ssr9 might counteract Asr9, keeping *tnp* expression low.

## INTRODUCTION

Insertion sequences (ISs) are small, mobile genetic elements that only carry the information required for their own transposition, i.e. a gene coding for a transposase and, commonly, a regulatory element to control the latter’s expression (1). They are grouped into families, largely on the basis of their transposase type, their similarity to other IS sequences and the presence of regulatory elements (2). While most ISs show low or no sequence specificity in their target selection, some insert only at specific sites with a particular DNA secondary structure or sequence. Upon insertion, ISs often generate a short direct repeat at both ends of the target sequence.

ISs are found both in plasmids and chromosomes, and can contribute significantly to the evolution of bacterial genomes, generating gene rearrangements (deletions or inversions of large DNA regions), mutations or changes in the expression of neighboring genes (1,3). In addition, ISs can generate composite transposons that facilitate the movement of genes conferring phenotypic traits to the host, such as virulence or resistance to antibiotics or toxic compounds. ISs are therefore important for the rapid evolution and adaptation of bacterial cells to new environments and stress conditions. However, transposition and DNA rearrangements can exert a considerable mutagenic burden on the host genome, so the expression of transposases is usually tightly regulated to ensure low transposition frequencies. Such regulation may involve the control of transcription and translation, the stability of the transposase mRNA, or the activity of the transposase (reviewed in 4–6).

**Figure 1.**
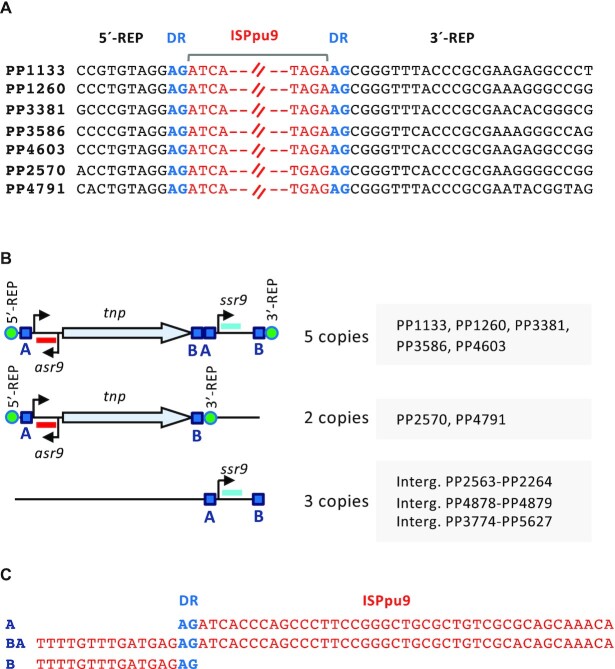
Diagram of ISPpu9 and its insertion sites in the *Pseudomonas putida* KT2440 genome. (**A**) Insertion site of each of the seven ISPpu9 copies according to (12). The sequence of the REP element interrupted by the ISPpu9 insertion is indicated in black font. The left and right ends of ISPpu9 are in red, and a direct repeat (DR) flanking the insertion site is indicated in blue. (**B**) Diagram of the two ISPpu9 variants (with or without the *ssr9*) and of the three orphan *ssr9* copies present in KT2440 genome. The transposase gene (*tnp*), the *asr9* (in red) and *ssr9* (blue) sRNAs identified in this work, and their respective promoters (arrowheads, as determined in Figure 2) are indicated. The DRs flanking the insertion site are represented by a green circle. The conserved ‘A’ and ‘B’ boxes identified (see text for details) are shown as blue squares. (**C**) Alignment of the ‘A’ and ‘B’ boxes. The AG direct repeat (DR) is indicated in blue.

*Pseudomonas putida* KT2440 is a soil bacterium that, due to its lack of virulence traits, its resistance to harsh conditions, and its robust and versatile metabolism, is an organism of choice for many biotechnological applications (7–9). Hence it has been thoroughly studied. The genome of this strain is 6.2 Mb in length (10) and contains 36 IS elements, most of them present in multiple copies (11). One of these ISs, named ISPpu9, is present as seven copies scattered throughout the chromosome. All seven are inserted at a specific site located within short conserved sequences that belong to the so-called repetitive extragenic palindromic (REP) family (10,12; see Figure 1A). REPs are highly conserved inverted repeats with the potential to generate stem-loop structures. They are found mostly in non-coding (extragenic) regions either as single units, or forming pairs or clusters of up to five elements. They are present in many bacterial species, although with a different sequence in each case (13–15). *P. putida* KT2440 has > 900 REPs with a highly conserved 35 bp sequence (14). It has been proposed that REPs are the remnants of the so-called REPINs, non-autonomous mobile genetic elements consisting of two REP sequences in inverted orientation separated by a spacer (16,17). The mobilization of these elements would require the use of an external transposase of the RAYT (REP-Associated tYrosine Transposase) family (18). REP sequences can recombine with one another generating chromosomal rearrangements (19), and are targets for some IS elements—such as ISPpu9—in a number of bacterial species (12,20). They can downregulate the translation of an upstream gene in some cases (21,22) and, when transcribed together with that upstream gene, form stable stem-loop structures at the 3′-end of the mRNA, increasing its stability (23,24).

**Figure 2.**
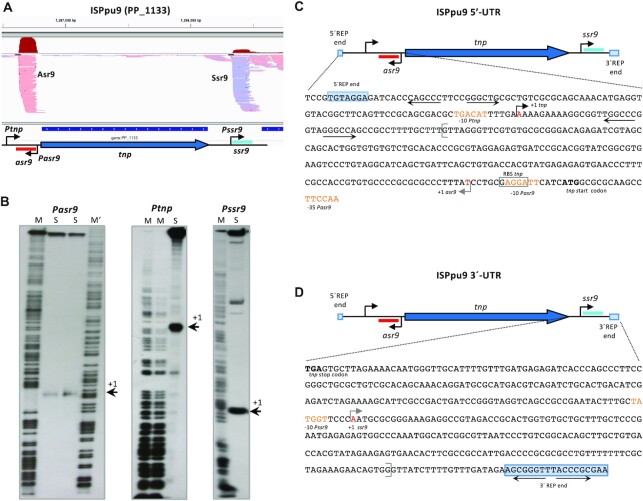
Identification of the 5′-ends of the *tnp*, *asr9* and *ssr9* RNAs. (**A**) Profile of the RNA reads recorded in a previously published RNA-Seq analysis of *P. putida* KT2440 (25) for the region corresponding to one of the ISPpu9 copies (PP_1133). ISPpu9 is depicted as in Figure 1B, and the observed RNA reads are indicated (light red for *asr9*, light blue for *ssr9*; the accumulated number of reads for each one is indicated on top in dark red), as observed using Integrative Genomics Viewer software (80). (**B**) Identification of the transcription start sites for *asr9*, *tnp* and *ssr9*, using S1 nuclease protection assays; the size of the protected labeled cDNA fragment complementary to each of the 5′ RNA region was determined by denaturing polyacrylamide gel electrophoresis, using a DNA sequence ladder (indicated as M or M’); the cDNA fragment is indicated by an arrow (+1). The sequence of the 5′- and 3′-ends of ISPpu9 are indicated in panels (**C** and **D**), respectively. The transcription start sites for *asr9*, *tnp* and *ssr9* are indicated by arrows; position + 1 is in red font. Sequences showing similarity to the -10 and -35 promoter elements recognized by sigma-70 RNA polymerase are indicated in orange font. The translation start site (ATG) for *tnp* is indicated in bold.

ISPpu9 contains a single ORF that codes for a DEDD-type transposase belonging to the poorly characterized IS*110*/IS*492* family (Figure 1B). The ISs associated with this family differ from those of other families in that they do not show clear inverted repeats at their termini and do not necessarily generate direct repeats at the insertion point (reviewed in 1). Analysis of the transcripts detected in a previously published RNA-Seq analysis of *P. putida* KT2440 (25) suggests that the gene coding for the ISPpu9 transposase (*tnp*) might be associated with two sRNAs, one encoded downstream of *tnp* on the same DNA strand, and the other immediately upstream of *tnp* on the complementary strand (Figure 2A). Antisense sRNAs complementary to the *tnp* mRNA have been observed for several transposons, although their involvement in regulating the translation or stability of the transposase mRNA has been little analyzed (reviewed in 6).

Despite the clear importance of ISs in the evolution of bacterial genomes, in horizontal gene transfer and in gene expression, little is known about how the expression of transposase genes is regulated. This report characterizes two sRNAs associated with ISPpu9 and analyses their participation in regulating the expression of the transposase gene. The results show that, despite the apparent simplicity of ISPpu9, the expression of its *tnp* gene is tightly controlled by a mechanism involving two partially complementary sRNAs and the *tnp* mRNA 5′-untranslated region (5′-UTR). To our knowledge, the participation of two sRNAs in the regulation of an IS has not been reported before.

## MATERIALS AND METHODS

### Bacterial strains and culture media

The *Escherichia coli* strains used in this work were DH5α (26), HB101 (pRK600) (27), CC118λpir (28) and BL21(DE3)-Δ*hfq::cat* S1, which is an Hfq-null derivative of the BL21(DE3) T7 expression strain (29). All *E. coli* strains were cultured in Lysogeny Broth (LB) (30) at 37°C. *P. putida* strains KT2440 and F1 have been previously described (31,32); these were cultured at 30°C in either LB or in M9 minimal salts medium (30) supplemented with trace elements (33) and 30 mM citrate as the carbon source. Antibiotics—kanamycin (50 μg/ml), ampicillin (100 μg/ml), chloramphenicol (50 μg/ml) and streptomycin (50 μg/ml)—were added as required. Cell growth was monitored by measuring turbidity at 600 nm.

To obtain *P. putida* F1 or KT2440 strain derivatives overproducing Asr9 or Ssr9, the *asr9* and *ssr9* genes, including their own promoters, were cloned into the broad-host-range plasmids pSEVA431 (medium copy-number pBBR1 replicon, ∼30 copies/cell in *P. putida*) and pSEVA451 (high copy-number RSF1010 replicon, ∼130 copies/cell in *P. putida*) (34,35). The *asr9* and *ssr9* sequences used were obtained by PCR-amplification with the primers specified in Supplementary Table S1 and cloned into plasmid pGEM-T Easy (Promega). The plasmids obtained were named pGEMT*asr9* and pGEMT*ssr9*, respectively. DNA fragments containing *asr9* or *ssr9* were excised from pGEMT*asr9* or pGEMT*ssr9* with EcoRI and cloned at the same restriction site of plasmids pSEVA431 and pSEVA451, obtaining plasmids p431-asr9, p431-ssr9, p451-asr9 and p451-ssr9, respectively. These plasmids were introduced into *P. putida* strains KT2440 and F1 by conjugation, as previously described (36), using plasmid pRK600 as the donor of transfer functions.

A *P. putida* F1 derivative containing *asr9* was constructed by excising a NotI DNA fragment containing *asr9* from plasmid pGEMT*asr9* and cloning it at the NotI site of plasmid pUT-mini-Tn5Sm (28), generating pTn5-*asr9*. This suicide plasmid was used to deliver the mini-Tn5Sm-*asr9* to the strain F1 chromosome by conjugation and streptomycin-resistant clones then selected. A representative clone was chosen and named F1-asr9. A similar strategy, using plasmid pGEMT*ssr9*, allowed the construction of strain F1-ssr9, which bears a copy of the *ssr9* sRNA in its chromosome.

### Northern blots

Total RNA was purified from cells growing exponentially in LB using the TRIzol Max Bacterial Isolation Kit (Ambion). DNA was eliminated using RNase-free DNase I (Roche) following the manufacturer’s instructions. RNA was treated with phenol/chloroform, ethanol precipitated and its integrity determined by agarose-gel electrophoresis.

Northern blots were performed essentially as previously described (37). Briefly, 5 μg of total RNA from the indicated strains were resolved by electrophoresis in a 7 M urea 6% polyacrylamide gel and transferred to a Nylon Hybond N + membrane (GE Healthcare Biosciences) using a semi-dry transfer unit. Membranes were UV-crosslinked and hybridized to a mixture of DIG-labeled probes directed against each sRNA and the 5S RNA. Bands were detected using the DIG Luminescent Detection Kit (Roche) and visualized by exposure to Agfa X-ray films or with a Chemidoc (BioRad). The latter was used to quantify band intensity.

To obtain digoxigenin-labeled RNA probes complementary to Asr9 or Ssr9 sRNAs, a DNA segment including *asr9* or *ssr9* was PCR-amplified using the primer pairs asr9A-D/asr9A-R or ssr9A-D/ssr9A-R (Supplementary Table S1), which include restriction sites for EcoRI and HindIII endonucleases. The resulting DNA fragments were digested with these enzymes and cloned into the corresponding sites of plasmid pSPT18 (Roche), obtaining plasmids pSPT18-asr9N and pSPT18-ssr9N, respectively. These plasmids were linearized with HindIII and used as templates for *in vitro* transcription reactions using the DIG RNA labeling kit (Roche) and T7 RNA polymerase. A probe against the 5S ribosomal RNA was obtained following the same procedure, using plasmid pSPT18-5S (37), which contains the 5S rRNA gene from *P. putida* KT2440, as a template.

### Identification of the transcription start sites by S1 nuclease protection assays

Total RNA was isolated as described for northern blots. S1 nuclease protection assays were performed as previously described (38), with small modifications. Briefly, a dsDNA fragment presumed to include the promoter of interest was PCR-amplified using *P. putida* KT2440 chromosomal DNA and the primer pairs indicated in Supplementary Table S1; the reverse primer had been previously labeled at its 5′-end using [γ ^32^P]-ATP and T4 polynucleotide kinase. The end-labeled DNA fragment obtained (50 000 cpm) was mixed with 25 μg of RNA obtained from *P. putida* KT2440, precipitated with ethanol, and resuspended in 80% formamide, 40 mM Pipes, pH 6.4, 400 mM NaCl, 1 mM EDTA. The mixture was incubated at 65°C for 10 min, and the RNA and DNA allowed to hybridize overnight at 37°C. Single stranded DNA regions were digested with 40 Units of S1 nuclease for 60 min at 30°C. The undigested DNA was ethanol-precipitated and analyzed by electrophoresis in a denaturing 6% urea-polyacrylamide gel, in parallel with a DNA sequence ladder obtained by chemical sequencing (39) of the same end-labeled DNA fragment.

### Reporter transcriptional and translational fusions and β-galactosidase activity assays

To obtain transcriptional fusions of promoters *Ptnp*, *Pasr9* or *Pssr9* to the *lacZ* reporter gene, DNA fragments containing each of these promoters were PCR-amplified using *P. putida* KT2440 genomic DNA as a template and the oligonucleotide pairs indicated in Supplementary Table S1. The amplified DNA segments were cloned between the BamHI and HindIII sites of plasmid vector pSEVA225 (40), which contains a promoterless *lacZ* gene downstream of the HindIII site. The reporter plasmids obtained were named pTNC-Ptnp, pTNC-Pasr and pTNC-Pssr, respectively. The *Ptnp-lacZ* fusion spanned positions -82 to + 235 relative to the *tnp* transcription start site, which includes all but two of the ISPpu9 nt located upstream of the transcription start site, and all the 5′-UTR up to the start of the ribosome binding site (RBS; the *tnp* translation start site is at position + 245). The *Pasr9-lacZ* and *Pssr9-lacZ* fusions included DNA segments spanning positions -286 to + 40 and -170 to + 12 relative to the transcription start site of each promoter, fused to *lacZ*.

Several translational fusions of the *tnp* gene to the ‘*lacZ* reporter gene were constructed. In all cases, the 5′ end was the same as indicated for the transcriptional fusions, while the 3′-end included either the first 2 or the first 8 codons of *tnp*, fused in-frame to the start of ‘*lacZ*. To construct them, appropriate DNA segments were PCR-amplified from *P. putida* KT2440 genomic DNA using the primer pairs cmFus-D and 2C-R, or cmFus-D and 8C-R (Supplementary Table S1), and the DNA fragments obtained cut with BamHI and HindIII and cloned between the corresponding restriction sites of plasmid pSEVA225T (40). The reporter plasmids generated were named pTNL-tnp2 and pTNL-tnp8 respectively. Five variants of these plasmids were made. Plasmids pTNL-tnp2S and pTNL-tnp8S were derived from pTNL-tnp2 and pTNL-tnp8, respectively, by inserting the *ssr9* gene at a SpeI site located downstream of the ‘*lacZ* gene. The *ssr9* sequence used was obtained from plasmid pGEMT*ssr9* as a SpeI fragment. Plasmid pTNL-tnp8m35 is similar to pTNL-tnp8 but contains five mutations at the -35 region of promoter *Pasr9* (the sequence 5′-CCTTCCAAGCAA was changed to 5′-CC**GAGT**AA**A**CAA; substitutions in bold). To obtain it, a DNA fragment including the entire ISPpu9 with the modifications indicated above was chemically synthesized (GenScript), and the sequences spanning promoter *Ptnp* and the first 8 codons of the *tnp* gene PCR-amplified with primers cmFus-D and 8C-R, which contain targets for BamHI and HindIII (Supplementary Table S1). The DNA fragment obtained was digested with BamHI and HindIII and cloned between the corresponding sites of plasmid pSEVA225T. Finally, plasmid pTNL-tnp2HL, which contains the first 2 codons of *tnp* fused in-frame to ‘*lacZ*, plus several point mutations in the 5′-UTR that render its secondary structure less complex, was chemically synthesized (GenScript). This synthesized fragment has restriction sites for BamHI and HindIII at its ends, and these were used to clone it into plasmid vector pSEVA225T. All plasmid constructs were sequenced to verify the absence of undesired mutations.

The reporter plasmids were introduced into *P. putida* strains KT2440, F1 or F1-*asr9*. For transcriptional fusions, β-galactosidase activity was assayed using *o*-nitrophenyl-β-D-galactoside (ONPG) as a substrate, as previously described (41). Since the activity of the translational fusions was low, the chemiluminescent substrate Galacton-Plus (from the Galacto-Light Plus Reporter Gene Assay System [Applied Biosystems]) was used instead, normalizing the luminescence units to the turbidity of the samples as previously described (42,43). Luminescence was measured in microtitre plates using a TECAN Infinite m200 reader equipped with a luminescence detector. At least three independent assays (biological replicates), each with three technical replicates, were performed.

### Real time reverse-transcription PCR

Total RNA was obtained from three independent cultures (biological replicas) as indicated above but with an extra DNase I treatment using the Turbo DNA-free kit (Invitrogen). The RNA obtained was transformed into cDNA using the cDNA Archive Kit (Applied Biosystems). Real-time PCR (RT-qPCR) was performed using the 2^–ΔΔCt^ comparative method (44), as previously described (45,46), employing primers directed toward *lacZ*, *rpoN*, *tnp*, *asr9* or *ssr9* (see Supplementary Table S1). The results were normalized relative to those obtained for *rpoN*, the expression of which remains constant under a wide range of growth conditions (47), or for the 5S rRNA, as specified.

### Purification of *P. putida* KT2440 Hfq protein

*Pseudomonas putida* proteins Hfq(6xHis), HfqY25D(6xHis) and HfqK56A(6xHis), encoded in the expression plasmids pVI2344, pVI2345 and pVI2346, respectively (29), were overproduced in the Hfq‐null strain *E. coli* BL21(DE3)-Δ*hfq*::*catS1* (29). The His-tagged proteins were purified using an Ni-NTA column as previously described (48).

### *In vitro* transcription and labeling of sRNAs

To obtain Asr9 and Ssr9 sRNAs by *in vitro* transcription, *asr9* and *ssr9* were PCR‐amplified with oligonucleotide pairs asr9-D and asr9-R and ssr9-D and ssr9-R respectively (Supplementary Table S1), and the resulting DNA fragments cloned between the EcoRI and HindIII sites of plasmid pSPT18 (Roche), obtaining plasmids pSPT-AS and pSPT-S. These plasmids were linearized with HindIII and used for *in vitro* transcription reactions using T7 RNA polymerase and [γ‐^32^P]‐UTP (3000 Ci/mmol) as described earlier (49). The RNA transcripts were resolved in an 8 M urea 8% polyacrylamide gel, eluted overnight in 0.5 M NH_4_AcO, 0.1% SDS and 1 mM EDTA, and precipitated with ethanol. Non-labeled RNA was synthesized in a similar way, but using non-radioactive NTPs. RNAs were dissolved in water, denatured at 95°C for 1 min, allowed to renature for 5 min on ice and stored at -20°C.

### RNA electrophoretic mobility shift assay

The binding of Hfq protein to Asr9 and Ssr9 was analyzed in binding reactions containing labelled RNA and purified Hfq (obtained as indicated in the previous sections) at the concentrations indicated in the figure legends, 1 μg of yeast tRNA and 10 U of RNasin RNase inhibitor (Promega), all in 20 μl of TBM buffer (45 mM Tris/HCl, pH 8.3, 43 mM boric acid, 3 mM MgCl_2_, 0.5% glycerol). After 20 min at 30°C, 4 μl of loading buffer (60% glycerol, 0.025% xylene cyanol) were added and the samples transferred to ice and loaded onto a 6% non-denaturing polyacrylamide gel. Electrophoresis was performed at 4°C at 150 V using TBM as the running buffer.

Hybridization of Asr9 to Ssr9 was analyzed by adding increasing concentrations of unlabeled Asr9 to a solution containing a fixed amount of radioactively labelled Ssr9, obtained as indicated above. Samples were denatured at 95°C for 1 min, placed on ice for 5 min, and incubated at 30°C for 30 min in 20 μl of binding buffer (20 mM Tris-HCl, pH 8.0, 1 mM MgCl_2_, 20 mM KCl, 10 mM Na_2_HPO_4_). Four microliters of loading buffer (60% glycerol, 0.025% xylene cyanol) were added and the samples resolved in a non‐denaturing 6% polyacrylamide gel as described above. Where indicated, purified Hfq protein was added to the binding reactions after denaturation of the sRNAs.

### Determination of Asr9, Ssr9 and *tnp* mRNA half-life

Cells were cultured in LB medium under vigorous aeration. At a turbidity of 0.5, rifampicin was added at a final concentration of 300 μg/ml to stop transcription (a concentration >6-fold that needed to stop cell growth). Culture was continued and, at different times, aliquots withdrawn, incubated on ice for 5 min with RNA Protect Bacterial Reagent (Qiagen) and processed to obtain total RNA as indicated for northern blot assays. The amount of Asr9, Ssr9 and *tnp* mRNA in each sample was determined by RT-qPCR, using 5S rRNAs as an internal control. In the case of Asr9, stability was also analyzed using northern blots, as described above, employing 5S rRNAs as an internal control, with total RNA obtained from strains KT2440 and F1, the latter containing or lacking plasmids pTNL-tnp8 (fusion 8C), pTNL-tnp8S (fusion 8C + S) or p225T-*asr9*. To obtain plasmid p225T-*asr9*, a SpeI DNA fragment containing *asr9* was excised from plasmid pGEMTasr9 and cloned into the SpeI site of pSEVA225T.

### Transposition rate

The complete ISPpu9 sequence, including *asr9* and *ssr9*, was PCR amplified from *P. putida* KT2440 chromosomal DNA using primers ISPpu9-D and ISPpu9-R (Supplementary Table S1) and cloned into plasmid pGEM-T Easy (Promega), obtaining plasmid pGEM-ISPpu9. The insert was sequenced to assure the absence of undesired mutations. A kanamycin resistance determinant was PCR-amplified from plasmid pSEVA225 (34) using primers Km-D and Km-R (Supplementary Table S1), digested with XbaI and inserted at the XbaI site of plasmid pGEM-ISPpu9 (located downstream of the *tnp* gene and 58 nt upstream of promoter *Pssr9* transcription start site). The plasmid obtained (pGEM-ISPpu9-Km) was digested with NotI and the fragment containing ISPpu9-Km inserted at the NotI site of the suicide delivery vector pKNG101 (50), obtaining pKN-ISPpu9Km. This plasmid was used to transfer ISPpu9-Km to different bacterial strains by conjugation (see below).

Two variants of pKN-ISPpu9Km were constructed. One of these, pKN-ISPpu9KmΔ*ssr9*, lacked the *ssr9* gene. The second, pKN-ISPpu9KmΔ*tnp*, had a deletion within the *tnp* gene. Both were constructed by way of a three-step SOE-PCR procedure (51), using pKN-ISPpu9Km as a template. To delete *ssr9*, a first PCR run was performed using primers to amplify from the 5′-end of ISPpu9 to the *ssr9* transcription star site; this generated a DNA fragment that included the *tnp* gene, its 5′-UTR, and the kanamycin-resistance determinant, but lacked the downstream sequences (primers ISPpu9-D and Δssr-R; Supplementary Table S1). The second PCR run generated a fragment that spanned from the *ssr9* 3′-end to the end of ISPpu9 (primers Δssr-D and ISPpu9-R). The reverse primer used in the first reaction, and the forward primer of the second reaction, had complementary 5′-tails. The third PCR reaction used the two DNA fragments generated in the two former reactions as templates, plus primers designed to amplify from the 5′-end of ISPpu9 through to its 3′-end (primers ISPpu9-D and ISPpu9-R, Supplementary Table S1). The amplified fragment was cloned into plasmid pGEM-T Easy, sequenced to ensure the absence of undesired mutations, excised with NotI from the resulting plasmid and inserted at the NotI site of plasmid pKNG101 to obtain pKN-ISPpu9KmΔ*ssr9*. A similar strategy was used to delete the *tnp* gene from pKN-ISPpu9Km; this involved the same external primer pairs as indicated above, plus the internal primers Δtnp-R (reverse primer, left ISPpu9 end) and Δtnp-D (direct primer, right ISPpu9 end; see Supplementary Table S1), finally obtaining pKN-ISPpu9KmΔ*tnp*.

Plasmids containing ISPpu9Km or its variants were introduced into *P. putida* strains by conjugation, using triparental filter mating assays and plasmid pRK600 as the donor of transfer functions, as previously described (36). After 5 h at 30°C, the cells were suspended in 1 ml of M9 medium, serially diluted in the same medium, and plated on to agar plates again containing M9 medium with citrate as the carbon source plus either kanamycin (selects for transconjugants) or no antibiotic (selects for all recipients, with or without ISPpu9-Km). This allowed the frequency of transconjugants per recipient to be determined. As a control, the number of Sm-resistant transconjugants was monitored; these represent outcomes in which the pKNG101-derived plasmids (which do not replicate in pseudomonads and carry an Sm-resistance determinant) had inserted into the chromosome by DNA recombination and not by transposition. Subtracting the number of the Sm-resistant transconjugants from the total provided the number of Km-resistant transconjugants in which transposition events into the chromosome had occurred.

## RESULTS

### The *tnp* gene has a long 5′-UTR and is flanked by two sRNAs

**Figure 3.**
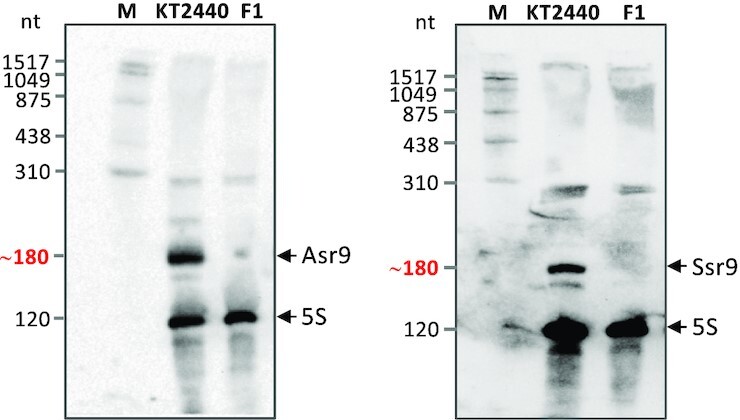
Identification of Asr9 and Ssr9 sRNAs by Northern blots. Total RNA from strains KT2440 and F1 was resolved in a denaturing polyacrylamide gel next to a DIG-labeled RNA size ladder (M), transferred to a membrane, and the sRNAs detected with probes for Asr9 (left) or Ssr9 (right), together with a probe for the ribosomal 5S RNA as control. The deduced approximate size for Asr9 or Ssr9 is indicated in red font.

The inspection of a recently published RNA-Seq analysis of *P. putida* KT2440 transcripts (25) showed the gene coding for the ISPpu9 transposase (*tnp*) to be expressed at very low levels (very few RNA reads were detected). Further, for five of the seven ISPpu9 copies, two short regions upstream and downstream of *tnp* accumulated a large number of reads (Figure 2A). The remaining two ISPpu9 copies showed upstream reads only. This suggests the presence of two sRNAs associated with ISPpu9. The precise transcription start site of these sRNAs, as well as that of the *tnp* gene, was determined in S1 nuclease protection assays (Figure 2B–D). The *tnp* mRNA had a long 5′-UTR (244 nt). The sRNA encoded upstream of *tnp* was complementary to the *tnp* 5′-UTR and was named Asr9 (antisense sRNA of ISPpu9), while that encoded downstream of *tnp* was transcribed in the same DNA strand as the *tnp* and was named Ssr9 (sense sRNA of ISPpu9; Figure 2). ARNold software analysis predicted stem-loop structures that might act as transcriptional terminators downstream of *asr9* and *ssr9* (52). These predicted terminators, plus the 3′-ends reported earlier in a genome-wide analysis of *P. putida* transcriptome (53), suggest both sRNAs to be close to 180 nt in length. Northern blots performed with RNA preparations from *P. putida* KT2440 revealed a ∼180 nt sRNA that hybridized to a probe for Asr9. The use of a probe for Ssr9 detected an sRNA of similar size (Figure 3). These two sRNAs were not detected in RNA samples obtained from *P. putida* strain F1, which lacks ISPpu9 (F1 was used as a control) (Figure 3). Relative-quantification by real-time RT-qPCR indicated that Asr9 was 4.6 ± 2.5 times more abundant than Ssr9.

Although two of the seven copies of ISPpu9 lack *ssr9*, inspection of the KT2440 chromosome identified three more copies of *ssr9* located in intergenic regions far from any ISPpu9 (Figure 1B). While the seven ISPpu9 copies are located at different sites in the KT2440 chromosome, they all lie at the same position in a REP sequence, generating an AG direct repeat (12; see Figure 1A). Interestingly, the first 41 nt downstream of the AG repeat at the 5′ end of the ISPpu9 copies were identical to a 41 nt segment located downstream of an AG dinucleotide present between the end of the *tnp* gene and the start of *ssr9* (blue squared boxes labeled ‘A’ in Figure 1B; the nt sequence is shown in Figure 1C). In addition, the 14 nt upstream of the AG dinucleotide located at the 3′-end of ISPpu9 copies containing *ssr9* were identical to a 14 nt sequence found downstream of *tnp* and immediately upstream of the 41 nt ‘A’ box mentioned above (blue squared box labeled ‘B’ in Figure 1B,C). The B and A boxes located downstream of *tnp* were connected by an AG dinucleotide (Figure 1C; ‘BA’ sequence). The three orphan *ssr9* copies were also flanked by A and B boxes, although for one of them the A-box included two mismatches. The sequences upstream and downstream of the insertion point of the three orphan *ssr9* genes were apparently not related to REP sequences. This arrangement of conserved sequences in ISPpu9, and the presence of ISPpu9 copies with or without *ssr9*, suggest that the mobilization of this IS might occur by transposition of an A-B segment including or lacking *ssr9*, although other alternatives cannot be ruled out.

### ISPpu9-like elements are present in other *P. putida* strains

ISPpu9 was initially described in strain KT2440 (12), but inspection of genome databases (https://www.pseudomonas.com;^54^) identified DNA sequences with 99.8–100% identity to the KT2440 ISPpu9 *tnp* gene in *Pseudomonas sp*. KBS0802 (seven copies) and *P. putida* NCTC13186 (seven copies). In all cases, sequences with 100% identity to KT2440 *asr9* were found immediately upstream of the *tnp* gene. Sequences corresponding to *ssr9* were detected downstream of some, but not all, of the *tnp* gene copies of these two strains (summarized in Supplementary Figure S1). In *Pseudomonas sp*. KBS0802, an *ssr9*-like sequence was present immediately downstream of five of the *tnp* genes, and in one an additional *ssr9*-like sequence followed in tandem. In the remaining two *tnp* copies, the *ssr9*-like sequence was absent. However, three independent *ssr9*-like copies were detected at other sites in the genome. For *P. putida* NCTC13186, an *ssr9*-like gene was present immediately downstream of six of the seven *tnp* copies and, in two, an additional *ssr9* gene was present in tandem. Further, four independent *ssr9* copies were found at different places in the genome, two of them in tandem. Strains KT2440, KBS0802 and NCTC13186 are closely related but not identical. The location and number of copies of *ssr9* clearly differ among these strains, suggesting that *ssr9* has some independence from, or can eventually detach from the *tnp* gene.

### Effect of Asr9 and the *tnp* 5′-UTR on the expression of *tnp*

**Figure 4.**
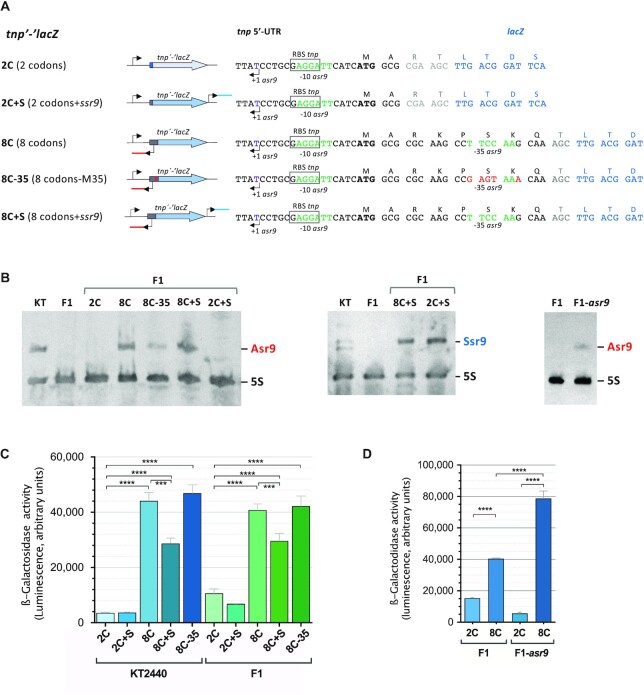
Influence of the 5′-UTR, Asr9 and Ssr9 on the expression of *tnp*, as determined by translational fusions to the ‘*lacZ* reporter gene. (**A**) Diagram showing the translational fusions to ‘*lacZ* constructed. The name of the fusion is on the left, followed by a diagram and a detailed description of the fusion region (right). Bent arrows indicate transcription start sites. The red and blue lines reporter construction diagram denote *asr9* and *ssr9* respectively, while the gray and blue regions of the thick arrow correspond to the first *tnp* codons (gray) and the ‘*lacZ* gene (blue). In the nucleotide sequences (right), positions in black font correspond to *tnp*, those in blue to ‘*lacZ*, and those in gray to extra codons introduced during cloning procedures. Positions mutated at the -35 region of promoter *Pasr9* are indicated in red font (construction 8C-35). (**B**) Presence or absence of Asr9 or Ssr9 in strains KT2440, F1, F1-*asr* or F1 containing the plasmids that include the translational fusions indicated in (A), determined by northern blots. (**C** and **D**) Levels of β-galactosidase observed in cells of strains K2440, F1 or F1-asr9 containing reporter plasmids including the indicated translational fusion (2C, 2C + S, 8C, 8C + S or 8C-35; see panel ‘A’ for a description of each fusion). Values represent the mean and the standard deviation of three biological replicates; cells were collected in mid-exponential phase (*A*_600_ of 0.5). Asterisks indicate significant differences between sample pairs specified by brackets (one-way ANOVA; ****P< 0.0001; ***P< 0.001).

The antisense Asr9 sRNA is complementary to a large part of the 5′-UTR of *tnp* mRNA, and its hybridization with the 5′-UTR could interfere with *tnp* mRNA translation. To explore this, several plasmids containing translational fusions of *tnp* to the ‘*lacZ* reporter gene were constructed that included or excluded the promoter for *asr9*. A *tnp’-‘lacZ* translational fusion, named 8C was constructed to include the first eight codons of *tnp*, which covers the complete promoter for *asr9* (*Pasr9*); it was therefore expected to produce Asr9 sRNA (see Figure 4A). Another fusion, named 2C, similar to the former was also constructed but included only the first two *tnp* codons; it therefore lacked the *Pasr9* -35 region, rendering it unable to produce Asr9 (Figure 4A). An additional fusion was constructed containing the first eight *tnp* codons but with five point-mutations in the -35 region of the promoter *Pasr9*; these were expected to render it inactive (fusion 8C-35; Figure 4A). The -10 region could not be modified since it overlaps the *tnp* ribosome binding site (RBS) (Figures 2C and 4A). The plasmids containing these three fusions were introduced into *P. putida* strains KT2440 and F1. Since strain KT2440 contains Asr9 and Ssr9 produced from the ISPpu9 copies present in its chromosome, it does not allow an analysis of whether the translational fusions constructed produce Asr9 or not. Strain F1, however, lacks ISPpu9 and *asr9*, and is therefore useful for monitoring whether the reporter plasmids can produce Asr9. Northern blots showed that the translational fusion containing the first eight *tnp* codons (fusion 8C) did produce Asr9 in strain F1, while the fusion containing only two codons (fusion 2C) did not (Figure 4B). However—and contrary to initial expectations—fusion 8C-35 still produced Asr9, indicating that the mutations introduced did not completely impair promoter *Pasr9*.

In strain F1, the activity recorded for the 2C fusion was about one quarter that observed for the 8C fusion (Figure 4C). The amount of ‘*lacZ* transcripts detected by relative-quantification real-time RT-qPCR for the 8C fusion was 1.41 ± 0.04 times that seen for the 2C fusion, a difference too small to explain the differences in β-galactosidase activity recorded for the two fusions. This difference is thus likely to derive from the translation, not transcription, of the reporter gene. When introduced into strain KT2440, which expresses ample amounts of Asr9 and Ssr9 from the ISPpu9 copies present in its chromosome, the activity observed for the 2C fusion was one twelfth that seen for the 8C fusion (Figure 4C). Finally, the fusion containing eight codons and a modified *Pasr9* promoter (fusion 8C-35) showed an activity similar to that of the wild-type *Pasr9* promoter, a result consistent with the observation that 8C-35 can still produce Asr9 (Figure 4B,C).

A key difference between the two reporter constructions is that fusion 8C allows the expression of Asr9, while fusion 2C does not (see Figure 4B). Since Asr9 is complementary to the 5′-UTR of *tnp* mRNA, the lesser activity of the 2C fusion compared to the 8C fusion suggests that Asr9 might facilitate the translation of *tnp* mRNA. The results obtained might therefore be explained by assuming that the access of ribosomes to the *tnp* mRNA is hindered by a strong secondary structure in the 2C fusion, a structure that would be unfolded in the mRNA produced by the 8C fusion due to hybridization with Asr9. However, the low activity of the 2C fusion in strain KT2440 is puzzling since Asr9 is present in this strain. A possible explanation is that these Asr9 copies are not available to the 2C fusion, perhaps because they fold into a stable secondary structure that hinders hybridization to *tnp* mRNA, or because they are sequestered via hybridization with Ssr9, to which they show partial complementarity (see below). The 8C fusion, however, can supply additional copies of Asr9 at a high local concentration that might overcome these problems.

In a further attempt to investigate the role of Asr9 in *tnp* translation, the *asr9* gene was inserted into the chromosome of strain F1 via a mini-Tn*5* delivery plasmid. The resulting strain, named F1-*asr9*, did produce Asr9 as detected by Northern blotting, and therefore allowed the effect of Asr9 in the absence of Ssr9 to be examined (Figure 4B). The plasmids containing the 2C or 8C reporter fusions were individually introduced into strain F1-*asr9*, and the β-galactosidase produced was analyzed in parallel to that generated by the same reporter plasmids in the parental strain F1. As shown in Figure 4D, the activity recorded for the 8C fusion in strain F1-*asr9* was double that seen for strain F1. Therefore, the Asr9 present in strain F1-*asr9* can increase translation of the 8C fusion, which is consistent with a stimulatory role for Asr9 in the translation of *tnp* mRNA. However, this stimulatory effect was not seen when the 2C fusion was tested, suggesting that Asr9 cannot properly hybridize with the mRNA produced by the 2C fusion. The reason might lie in the sequence between codons 2 and 8, which are missing in the 2C fusion and might be important for Asr9 activity, for example by providing an anchor point at which hybridization can start.

**Figure 5.**
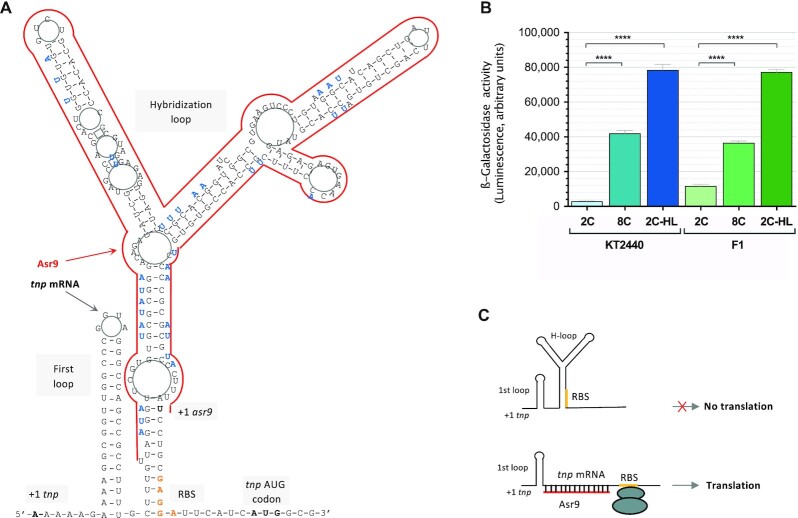
(**A**) Predicted folding of the 5′-UTR of *tnp* mRNA, as deduced by RNAFold software (78) at http://rna.tbi.univie.ac.at/cgi-bin/RNAWebSuite/RNAfold.cgi. The red line represents the region of the 5′-UTR complementary to Asr9 sRNA. The loops referred to in the text as ‘first loop’ and ‘hybridization loop’ are indicated, as well as the *tnp* and *asr9* transcription start sites (+1), the ribosome binding site for *tnp* mRNA (orange), and the *tnp* AUG codon (bold). In blue font, and adjacent to the wild type sequence, mutations introduced into the construct 8C-HL to weaken the hybridization loop. (**B**) Levels of β-galactosidase observed in strain KT2440 or F1 transformed with the reporter plasmids containing the translational fusions 2C (includes the first 2 codons of *tnp* fused in-frame with *‘lacZ*; does *not* express *asr9*; see Figure 4), 8C (8 codons of *tnp* fused in-frame to *‘lacZ*; does express *asr9*) or 8C-HL (same as 8C but with mutations at the hybridization loop designed to weaken the secondary structure). Asterisks indicate significant differences between the values recorded for reporter fusions 8C and 8C-HL relative to fusion 2C (one-way ANOVA; ****P< 0.0001); three biological replicates were performed. (**C**) Scheme representing how pairing of *asr9* to the 5′-UTR of *tnp* mRNA could unfold the hybridization loop, making the RBS accessible to ribosomes and allowing translation.

Finally, to further examine whether the *tnp* 5′-UTR folds into a secondary structure that inhibits *tnp* translation, an additional reporter fusion named 2C-HL was made. This construct was similar to the 2C fusion but included several point mutations at the 5′-UTR designed to weaken the secondary structure predicted to bury the RBS (and therefore hinder translation) (see Figure 5A). These point mutations were therefore expected to improve translation. When introduced into strain KT2440, the activity of the 2C-HL reporter fusion was 26 times that of the 2C fusion (Figure 5B). In strain F1, the activity of the 2C-HL fusion reached the same level as in KT2440. This again supports the idea that the *tnp* mRNA 5′-UTR forms a strong secondary structure that buries the RBS and impairs translation, and that unfolding this secondary structure, for example by pairing with Asr9, improves translation (Figure 5C).

### Effect of Ssr9 sRNA on the expression of *tnp*

**Figure 6.**
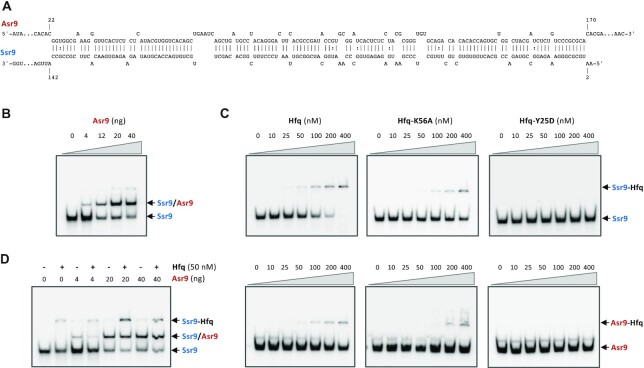
Hybridization of Asr9 to Ssr9. (**A**) Regions of sequence complementarity in Asr9 and Ssr9; alignment was performed using intaRNA software (79) at http://rna.informatik.uni-freiburg.de. (**B**) Increasing amounts of unlabelled Asr9 were mixed with a fixed amount of radioactively labeled Ssr9, and the complexes formed were resolved by non-denaturing gel electrophoresis. The presumed Ssr9/Asr9 complexed formed is indicated. (**C**) Binding of Hfq protein to Ssr9 and Asr9. Increasing amounts of purified Hfq (0–400 nM) were added to labeled Ssr9 or Asr9, and the complexes formed were resolved by non-denaturing gel electrophoresis. (**D**) Effect of Hfq on the formation of an Asr9/Ssr9 hybrid. Reaction mixtures were prepared as in (B) but in the absence or presence of purified Hfq protein (50 nM).

If the expression of the transposase needs to be kept in check to avoid excessive IS mobilization, and if Asr9 is indeed able to activate *tnp* translation, then its availability is likely to be controlled. This might be the role of Ssr9 sRNA. Ssr9 and Asr9 show extended regions of partial complementarity (Figure 6A). Therefore, Ssr9 might counteract Asr9 activity by sequestering it, acting like an ‘RNA sponge’, as reported for other sRNAs (55,56). This possibility was studied via both *in vitro* and *in vivo* experiments.

To analyze the effect of Ssr9 on *tnp* mRNA translation, a DNA fragment including *ssr9* and its corresponding promoter was introduced downstream of the ‘*lacZ* gene in the translational fusions 2C and 8C, rendering fusions 2C + S and 8C + S (Figure 4A). These fusions were introduced into strain F1 (which lacks Ssr9), and the correct expression of *ssr9* confirmed by northern blotting (Figure 4B). In strain F1, the levels of β-galactosidase generated by the 8C + S translational fusion (produces Asr9 and Ssr9) were 27% lower than those generated by the 8C fusion (contains Asr9 but lacks Ssr9). A similar trend was observed in strain KT2440 (35% reduction). With the translational fusion containing only the first two *tnp* codons, which does not produce Asr9, the presence of Ssr9 (fusion 2C + S) had no effect on β-galactosidase levels, as expected (Figure 4B). These results support the idea that Ssr9 can counteract the stimulating effect of Asr9.

### Ssr9 forms an RNA–RNA duplex with Asr9 *in vitro*

As mentioned above, Asr9 and Ssr9 show significant complementarity and could therefore form an imperfect RNA duplex (Figure 6A). To explore this possibility, the two sRNAs were overproduced *in vitro* and purified. In the production of Ssr9, [α^32^P]-UTP was incorporated into the transcription reaction to obtain a radioactively labeled RNA. A fixed amount of [α^32^P]-Ssr9 was then mixed with increasing amounts of unlabeled Asr9, and the RNAs denatured by heat and allowed to hybridize. The RNA species formed were resolved by non-denaturing polyacrylamide gel electrophoresis and those containing Ssr9 (the labeled ones) detected by autoradiography. As shown in Figure 6B, the addition of increasing amounts of unlabeled Asr9 led to the progressive disappearance of the band corresponding to Ssr9, while a new labeled band of lesser electrophoretic mobility appeared. Since Asr9 was unlabeled, the formation of the new band suggests that Asr9 can hybridize with Ssr9, forming an RNA-RNA duplex.

### Hfq binds to Asr9 and Ssr9 but has no influence on the formation of Asr9/Ssr9 hybrids *in vitro*

The Hfq RNA chaperone is known to bind many sRNAs and, in many cases, it facilitates their pairing with the target RNA (57,58). As shown in Figure 6C, purified *P. putida* Hfq protein was able to bind radioactively labeled Asr9 and Ssr9, although the protein’s affinity for Ssr9 (apparent dissociation constant 80 ± 10 nM) was 3.6 times that for Asr9 (apparent dissociation constant 291 ± 44 nM). *Escherichia coli* Hfq is a ring-shaped hexamer with two distinct RNA binding surfaces; the so-called proximal face which is required for the binding of U-rich RNAs, and the distal face that preferentially binds A-rich RNAs (59–61). *Pseudomonasputida* Hfq has the same properties (48,29). The use of *P. putida* Hfq derivatives bearing single amino acid substitutions either on the proximal (Hfq-K56A) or the distal face (Hfq-Y25D) showed that the Y25D substitution impaired the binding of Hfq to both sRNAs, while the K56A substitution did not, although the affinity of the protein was reduced. These results suggest that Hfq binds both sRNAs mainly through the distal face.

To investigate whether Hfq binding to Asr9 and Ssr9 might facilitate the hybridization of these two sRNAs, a fixed amount of labeled Ssr9 was incubated with increasing concentrations of unlabeled Asr9 in the absence or presence of 50 nM Hfq, and the appearance of RNA duplexes analyzed by electrophoresis. As shown in Figure 6D, Hfq had no visible effect on the formation of the RNA duplex under the conditions used. Neither higher nor lower Hfq concentrations changed this result (not shown).

### Asr9 is an unusually stable sRNA

**Figure 7.**
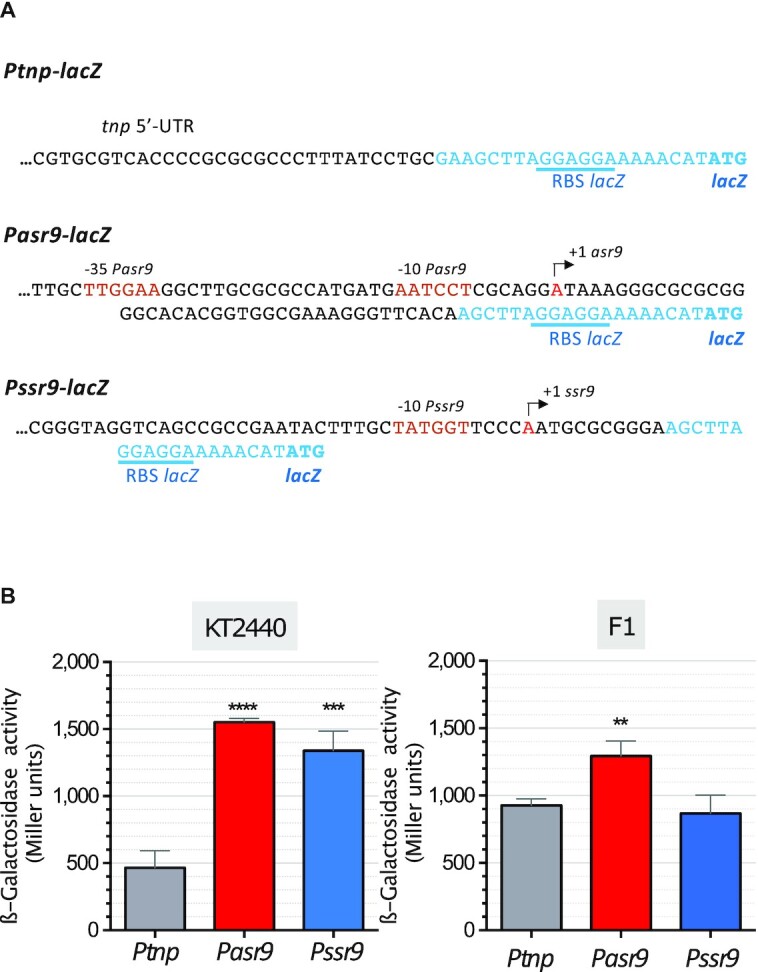
Activity of promoters *Ptnp*, *Pasr9* and *Pssr9*. (**A**) Transcriptional fusions to *lacZ* used to determine the activity of the indicated promoters; the sequences corresponding to *lacZ* are indicated in blue font. (**B**) The transcriptional fusions indicated in (A), cloned into a broad-host-range plasmid vector, were introduced into *P. putida* strains KT2440 and F1, and the β-galactosidase activity determined when cells reached a turbidity (*A*_600_) of 0.5. Values represent the mean and the standard deviation of three biological replicates. Asterisks indicate significant differences between the values recorded for *Pasr9* or *Pssr9* relative to *Ptnp* (one-way ANOVA; ****P< 0.0001; ***P< 0.001; **P< 0.01).

The RNA-Seq assays indicated Asr9 to be more abundant than the *tnp* mRNA, to which it pairs (Figure 2A). This could be due to the promoter for *tnp* gene having a smaller transcriptional output than the promoter for *asr9* or to strong differences in the stability of the respective RNAs. The activity of the promoters of *tnp* and *asr9* was analyzed with the help of transcriptional fusions to the *lacZ* reporter gene; for comparison, the activity of *Pssr9* promoter was analyzed as well. The reporter fusions (*Ptnp-lacZ*, *Pasr9-lacZ* and *Pssr9-lacZ*) were cloned into the broad-host-range plasmid pSEVA225 and introduced into strains KT2440 and F1. As shown in Figure 7, the transcriptional activity of promoters *Pasr9* and *Pssr9* in strain KT2440 was ∼3 times that seen for *Ptnp*. This difference was smaller in strain F1. Since the RNA-Seq assays showed that *tnp* mRNA was very low, while Asr9 and Ssr9 sRNAs were clearly detected (Figure 2A), the stability of the *tnp* mRNA is likely to be low.

**Figure 8.**
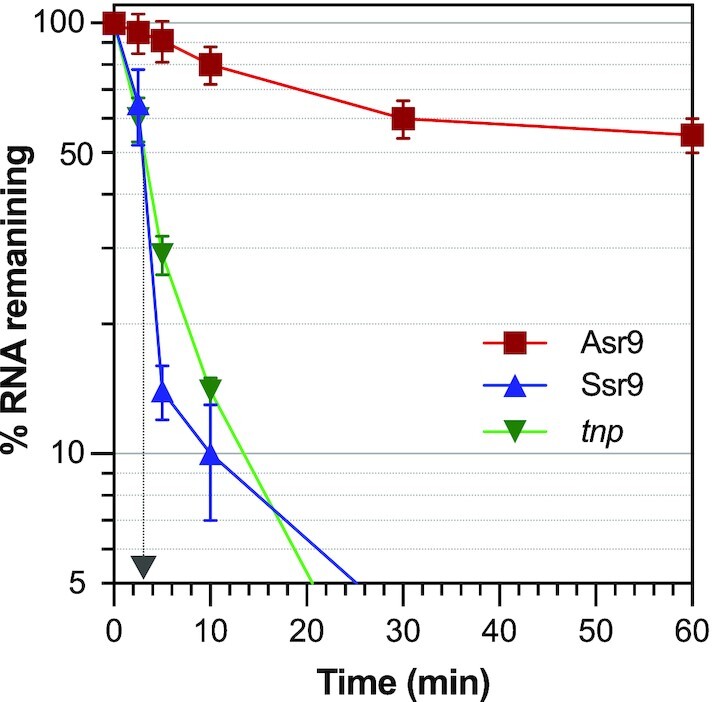
Stability of Asr9 and Ssr9 sRNAs, and of *tnp* mRNA. The amount of Asr9 (red line), Ssr9 (blue line) or *tnp* mRNA (green line) in KT2440 cells was determined by RT-qPCR at different times after blocking transcription by the addition of rifampicin. Values show the percentage of each RNA observed at the indicated times relative to time zero, normalized for the amount of 5S rRNA detected in each sample. Values correspond to the mean for three independent assays. The time at which only 50% of the initial amount of Ssr9 and *tnp* RNAs remained is indicated by an arrow (∼3 min).

**Figure 9.**
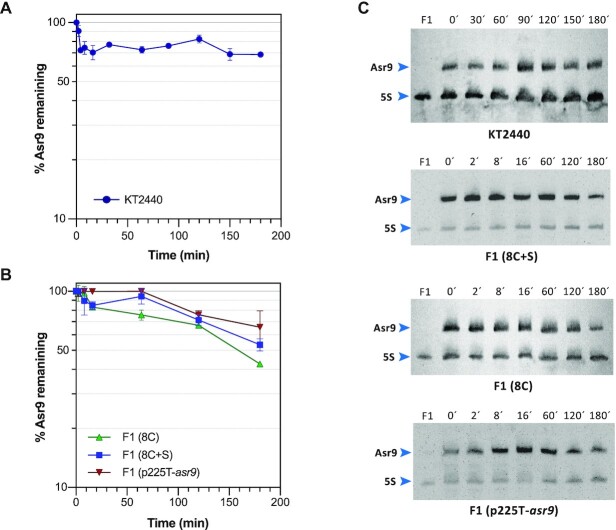
Influence of Ssr9 on Asr9 stability. The decay of Asr9 sRNA after blocking transcription by addition of rifampicin was determined by northern blots in RNA samples obtained from cultures of strains KT2440 (panel **A**), or F1 containing or lacking plasmids including the translational fusions 8C, 8C + S, or the *asr9* gene (panel **B**). Values indicate the percentage of Asr9 observed at each time relative to time zero, normalized for the amount of 5S rRNA detected (values correspond to the mean for three independent assays). (**C**) Examples of Northern blots used to monitor the decay of Asr9 in each strain. The time (in min) at which cell samples were collected after rifampicin addition is indicated. The well named ‘F1’ corresponds to RNA obtained from strain F1 (which does not contain Asr9), which was used as control. The bands corresponding to Asr9 and 5S rRNA are indicated.

The half-life of the Asr9 and Ssr9 sRNAs, and that of *tnp* mRNA, was determined by RT-qPCR after blocking transcription with rifampicin. The half-life of Ssr9 and *tnp* mRNA was ∼3 min, while that of Asr9 was >60 min (the last time point used in the assay) (Figure 8). The use of northern blots showed that, even after 180 min, some 70% of the initial amount of this sRNA was still detectable (Figure 9A). Most bacterial mRNAs have a half-life of 2 to 8 min (62). In the case of sRNAs, half-lives can vary from 2 to >30 min (63). The present results therefore indicate Asr9 to be a very stable sRNA.

The possible influence of Ssr9 and the *tnp* 5′-UTR on Asr9 stability was analyzed by introducing into *P. putida* F1 plasmids containing either the 8C + S translational fusion (generates the 5′-UTR, Asr9 and Ssr9), the 8C fusion (produces the 5′-UTR and Asr9, but not Ssr9) or just the *asr9* gene expressed form its own promoter (plasmid p225T-*asr9*). In the strain containing the 8C + S fusion, Asr9 half-life was ∼180 min, still a considerably long half-life, but shorter than that recorded for strain KT2440 (Figure 9). The absence of Ssr9 (fusion 8C) had a small impact on Asr9 stability, reducing its half-life to ∼160 min, while the lack of both the *tnp* 5′-UTR and Ssr9 (plasmid p225T-*asr9*) led to a small but detectable increase in Asr9 stability. Although strains KT2440 and F1 have different genetic backgrounds, and therefore comparisons should be made with caution, the results suggest that the stability of Asr9 derives from its overall sequence/structure, a stability that would decrease somewhat in the presence of Ssr9 and/or the *tnp* 5′-UTR.

### Influence of Asr9 and Ssr9 on the transposition efficiency of an incoming ISPpu9

The above results reveal the influence that Asr9 and Ssr9 can have on *tnp* expression once ISPpu9 has become established in the chromosome. The possible effect of these sRNAs on the transposition efficiency of new ISPpu9 copies entering the cell via conjugation was next analyzed.

**Figure 10.**
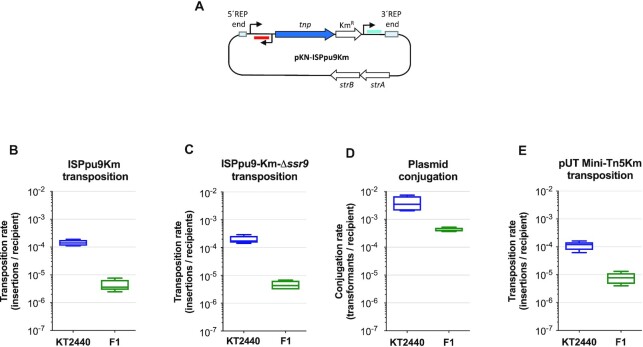
Transposition frequency of ISPpu9 in different genetic backgrounds. (**A**) Representation of plasmid pKN-ISPpu9Km, which contains the complete ISPpu9 insertion sequence and a kanamycin-resistant determinant inserted downstream of the *tnp* gene. Asr9 is represented in red, while Ssr9 is depicted in blue. (**B** and **C**) The pKN-ISPpu9Km suicide delivery plasmid, or its derivative lacking *ssr9* (pKN-ISPpu9KmΔ*ssr9*), were transferred by conjugation into strains KT2440 or F1, as specified in Materials and Methods section; the number of transposition events per recipient is indicated. (**D** and **E**) Plasmids pSEVA225T (control for conjugation; D) and plasmid pUT-Mini-Tn5Km (control for transposition; E) were transferred to strains KT2440 and F1 using the same procedure. Values are the average of five independent assays.

To measure transposition frequency, a gene specifying resistance to kanamycin (including a transcriptional terminator at its 3′-end) was inserted at the intergenic region between *tnp* and *ssr9*, and the construct cloned into the suicide delivery plasmid pKNG101, generating pKN-ISPpu9Km (see Figure 10A). This plasmid replicates in *E. coli* but not in *Pseudomonas*, and bears an Sm-resistance determinant as a marker. As a control, a similar plasmid was constructed in which the *tnp* gene encoding the transposase had been deleted (pKN-ISPpu9KmΔ*tnp*). Km-resistant transconjugants were then sought. Any arising could only be produced by (i) a transposition event placing ISPpu9 in the chromosome, which would render a Km-resistant but Sm-sensitive cell or (ii) by a DNA recombination event in which the complete plasmid is incorporated into the chromosome, resulting in cells resistant to both Km and Sm. When pKN-ISPpu9Km was delivered to strain KT2440 by conjugation, the number of Sm-resistant transconjugants was barely detectable (<8 × 10^–9^ per recipient), similar to the number of Km-resistant transconjugants obtained when the control plasmid lacking the transposase was used (pKN-ISPpu9KmΔ*tnp*). In contrast, plasmid pKN-ISPpu9Km consistently rendered ∼1.5 × 10^–4^ Km-resistant transconjugants per recipient (Figure 10B), a value four orders of magnitude above that observed in the control assays. When strain F1 was used as a recipient, plasmid pKN-ISPpu9Km rendered ∼4 × 10^–6^ insertions/recipient, which is one-fortieth that recorded for strain KT2440 (Figure 10C). This was not unexpected since strain KT2440 is a restriction-deficient strain while F1 is not (31); in fact, the use of the broad-host-range plasmid pSEVA225T to measure the conjugation rate showed that of strain F1 to be one eighth that of KT2440 (Figure 10D). An additional control was performed using the delivery plasmid pUTmini-Tn5Km (28), which enters *P. putida* by conjugation but is unable to replicate in this host, allowing transposition of the mini-Tn5Km transposon. In this case, the transposition frequency in strain F1 was one sixteenth that seen for KT2440 (Figure 10E).

**Figure 11.**
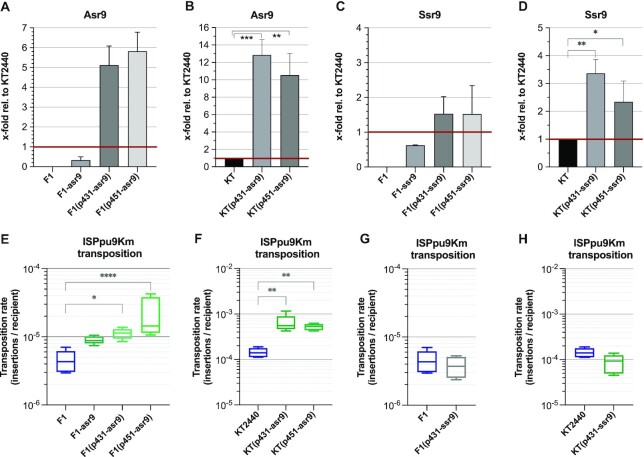
Effect of overproducing Asr9 and Ssr9 on the transposition frequency of ISPpu9. (**A–D**) Abundance of Asr9 (panels A, B) or Ssr9 (panels C, D) in strains KT2440, F1, F1-asr9 (contains one copy of *asr9* in the chromosome), or KT2440 and F1 containing plasmids p431-asr9, p451-asr9, p431-ssr9 or p451-ssr9. Values were obtained by comparative RT-qPCR and are expressed relative to the amount of Asr9 or Ssr9 detected in strain KT2440 (indicated as ‘1’ and highlighted with a red line). Three independent assays (with three technical replicas each) were performed; statistical significance was evaluated by one-way ANOVA. (**E–H**) Transposition frequency of ISPpu9Km when introduced by conjugation into the indicated bacterial strains containing variable amounts of Asr9 (panels E, F) or Ssr9 (panels G, H). Values are the mean of 5 to 7 independent assays. The differences in transposition frequency were analyzed using the non-parametric Kruskal–Wallis test. *P< 0.05; **P< 0.01; ***P< 0.001; ****P< 0.0001.

An important difference between strains KT2440 and F1 that might be related to the higher transposition rate of ISPpu9-Km in KT2440, is that when the IS enters KT2440, Asr9 and Ssr9 sRNAs are already present in the cell, while this is not the case in strain F1 (which lacks ISPpu9). To analyse whether the presence of Asr9 in the cell facilitates the transposition of an incoming ISPpu9, ISPpu9Km was delivered to strain F1 derivatives containing increasing amounts of ectopically expressed Asr9. These were constructed introducing either a single ectopic copy of *asr9* into the chromosome (strain F1-asr9) or a broad-host-range plasmid containing *asr9* expressed from its own promoter. Two plasmids were constructed which bear replication origins that typically provide ∼30 copies/cell (plasmid p431-asr9) or ∼130 copies/cell (plasmid p451-asr9) when propagated in *P. putida* (34,35). Comparative RT-qPCR showed that the Asr9 concentration in strain F1-asr9 was lower than in strain KT2440 (0.3 ± 0.1 times; see Figure 11A). The presence of plasmids p431-asr9 or p-451-asr9 led to Asr9 concentrations 5.1 ± 1 and 5.8 ± 1 times (respectively) those of strain KT2440 (Figure 11A). In strain F1-asr9, the transposition frequency of an incoming ISPpu9Km was twice that seen in strain F1 (Figure 11E), although this difference was barely significant (*P* = 0.1). The presence of plasmids p431-asr9 or p451-asr9 increased the transposition frequency by 3- (*P* = 0.01) or 3.3 times (*P* < 0.001), respectively (Figure 11E). When plasmids p431-asr9 and p451-asr9 were introduced into strain KT2440, the amount of Asr9 in the cell increased 10–12 times (Figure 11B); the transposition frequency increased as well (3.8 and 3.9 times, respectively; *P* = 0.005; Figure 11F). These results are consistent with the idea that Asr9 improves *tnp* mRNA translation, which would in turn increase transposase levels and transposition frequency.

As a first step in analyzing the influence of Ssr9 on transposition frequency, a variant of plasmid pKN-ISPpu9Km was constructed in which the *ssr9* gene was deleted; this was named pKN-ISPpu9KmΔ*ssr9*. The introduction of this modified delivery plasmid into strains KT2440 and F1 showed that the absence of *ssr9* had little impact, if any, on transposition efficiency of the incoming ISPpu9 (Figure 10C; compare with Figure 10B). Derivatives of strain F1 were then obtained containing either an ectopic *ssr9* copy in the chromosome (strain F1-ssr9), or the multicopy plasmids p431-ssr9 and p451-ssr9, which contain the *ssr9* gene. The Ssr9 concentration in strain F1-ssr9 was ∼0.6 times that of strain KT2440, while plasmids p431-ssr9 and p451-ssr9 increased this concentration to ∼1.5 times that of strain KT2440 (Figure 11C). These values are clearly lower than those obtained when overproducing Asr9, a result that probably derives from the much lower stability of Ssr9 compared to Asr9. The transposition frequency of ISPpu9 in strain F1 containing plasmid p431-ssr9 was similar to that observed in strain F1 (Figure 11G). When plasmid p431-ssr9 was introduced into strain KT2440, Ssr9 concentration tripled (Figure 11D) and a small reduction in transposition frequency was detected (*P* = 0.04) (Figure 11H).

## DISCUSSION

Knowledge of the regulation of ISs activity is important if we are to understand the stability of bacterial genomes. Despite the apparent simplicity of ISs, the few that have been studied in detail show that the expression of a given transposase can be controlled by more than one mechanism, and that different ISs rely on different ones. A simple and economic solution is to tightly regulate the translation of the transposase gene using an antisense sRNA. For example, for IS*10*, IS*30* and IS*200*, an antisense sRNA helps to reduce translation and/or the stability of the mRNA coding for the transposase (64–67; reviewed in 6). In some cases, the expression of the transposase is controlled by a host protein such as Hfq, or by both an sRNA and Hfq, as determined for IS*10*/Tn*10* (68,69), IS*200* (67) and IS*50*/Tn*5* (70).

Although most of the sRNAs described inhibit the translation of the target mRNA upon binding, in some cases they have the opposite effect, i.e. they stimulate translation (reviewed in 71). Translation stimulation can occur when the binding of the sRNA to the target mRNA either protects the mRNA from degradation by RNases (72–74) or changes its secondary structure making the RBS more accessible to ribosomes (75–77). In the case of ISPpu9, the results reported here suggest that the *tnp* 5′-UTR folds into a secondary structure that inhibits translation, most likely by making the RBS inaccessible to the translation initiation machinery. Mutations designed to weaken this putative secondary structure increased the activity of a *tnp’-‘lacZ* reporter translational fusion (Figure 5). The Asr9 antisense sRNA is complementary to the 5′-UTR sequence that appears to fold into a secondary structure to bury the RBS, and it is here proposed that the binding of Asr9 to *tnp* mRNA would unfold this inhibitory secondary structure, facilitating translation. Translational fusions containing *asr9* were up to 12 times more active than those lacking it, suggesting that this antisense sRNA can improve *tnp* translation (Figure 4). The observation that fusion 8C, which produces Asr9, was twice as active in strain F1-*asr9* as in the parental strain F1 (Figure 4D) shows that an increase in the amount of Asr9 available can increase *tnp* translation. Thus, the Asr9 antisense sRNA seems to have a stimulatory effect on *tnp* translation, which is the opposite to that observed for the antisense sRNAs of IS*10*, IS*30* or IS*200*, all of which inhibit the translation of the corresponding *tnp* mRNA (6).

When strain KT2440—which contains high levels of Asr9 and Ssr9 produced by the seven chromosomal copies of ISPpu9—was used as a recipient, the transposition frequency of an incoming (by conjugation) ISPpu9 element was of the order of 1.5 × 10^-4^ transformants per recipient. Introduction into strain KT2440 of a multicopy plasmid containing *asr9* increased the Asr9 concentration about 12 times and almost quadrupled the transposition frequency of an ISPpu9 element entering the cell by conjugation. This is consistent with an activating effect of Asr9 on *tnp* expression that leads to an increase in transposition frequency.

A similar effect was observed when Asr9 was overproduced in strain F1, which lacks Asr9/Ssr9 because it has no pre-existing copies of ISPpu9. In this strain, the transposition frequency was about one fortieth that seen in strain KT2440. Two reasons could explain this difference. First, plasmid conjugation in strain F1 was consistently one-eighth as efficient as in KT2440, possibly due to restriction barriers being stronger in F1. After correcting for the differences in conjugation efficiency, the transposition of ISPpu9 in strain F1 was about one fifth as efficient as in strain KT2440. The second reason derives from the absence of a pre-existing pool of Asr9 when ISPpu9 enters strain F1 for the first time, which contrasts with the ample amounts of Asr9 present in strain KT2440 generated from the 7 pre-existing copies of this IS present in the chromosome. Therefore, an ISPpu9 element that enters strain F1 likely attains a less efficient expression of the transposase gene than when it enters strain KT2440, which leads to lower transposition frequencies. The use of an F1 variant with a multicopy plasmid containing *asr9* allowed Asr9 to be produced at concentrations 5–6 times those seen in strain KT2440. In turn, this led to a tripling of the ISPpu9 transposition efficiency compared to the parental plasmid-free F1 strain, a value close to the transposition efficiency observed in strain KT2440 after correcting for the difference in conjugation frequency. This result further strengthens the conclusion that Asr9 stimulates both *tnp* expression and the transposition frequency.

**Figure 12.**
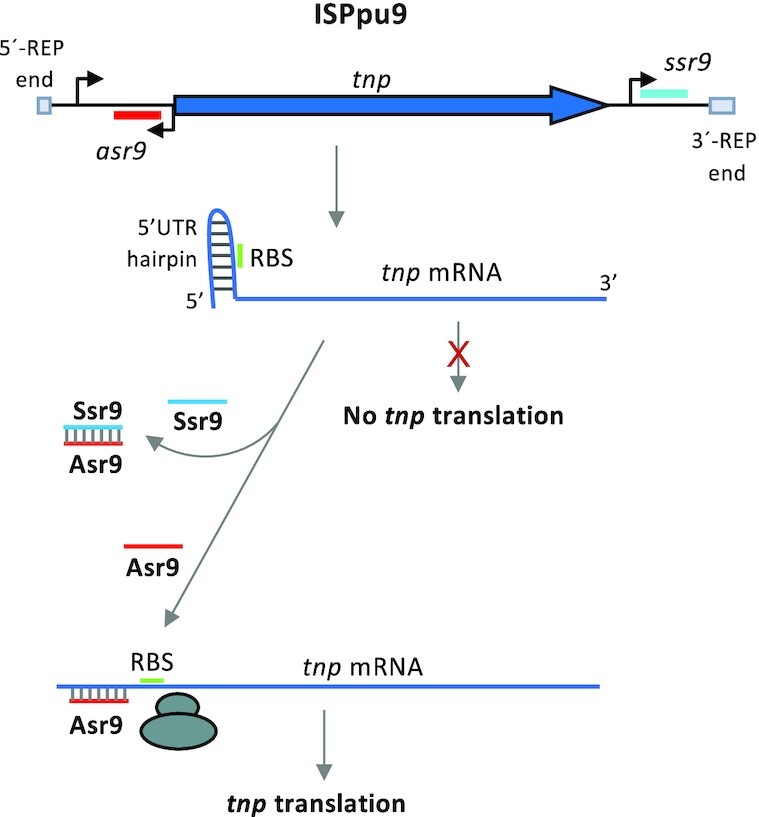
Proposed model for the regulation of ISPpu9 *tnp* expression. Diagram showing how Asr9 might facilitate the weakening of the secondary structure proposed to block the translation of *tnp* mRNA, and how Ssr9 might counteract this by binding to Asr9 and impeding its interaction with *tnp* mRNA.

The present work also provides evidence suggesting that the Ssr9 sRNA can antagonize Asr9. Ssr9 was able to hybridize with Asr9 *in vitro*, forming an RNA duplex (Figure 6), and the presence of Ssr9 reduced the stimulatory effect of Asr9 on *tnp* mRNA translation (Figure 4). The reduction detected, of ∼30%, is consistent with the observation that Ssr9 abundance was about 4–5 times lower that of Asr9. Asr9 was found to be a very stable sRNA. All these observations suggest that Ssr9 acts as an RNA sponge (56), hybridizing to Asr9 and hindering its binding to its target at the *tnp* mRNA 5′-UTR. Since Ssr9 stability was much lower than that of Asr9, and its concentration was 4–5 times lower that of Asr9, it is likely that the Asr9/Ssr9 hybrid is quickly degraded. The regulatory mechanism proposed is summarized in Figure 12. To our knowledge, at this time there are no other examples of two sRNAs directly participating in controlling the expression of an IS transposase. The need for the second sRNA likely derives from the stimulatory effect of Asr9 on *tnp* mRNA translation and its high stability that would require a mechanism to reduce Asr9 concentration and keep *tnp* expression in check to ensure low ISPpu9 activity once it has inserted into the chromosome (thus minimizing any mutagenic burden). In addition, Ssr9 might facilitate the degradation of Asr9, which is very stable and accumulates in the cell. It is interesting that the translational fusions containing or lacking *asr9* and/or *ssr9* behaved similarly in strains KT2440 (which contains Asr9/Ssr9) and F1 (which lacks previous copies of Asr9/Ssr9). This suggests that a large part of the Asr9 sRNA in strain KT2440 is not functional, perhaps because it is sequestered by Ssr9 or because it has acquired a stable secondary structure that has a low tendency to pair with the *tnp* 5′-UTR.

The final outcome of the regulatory strategy described is that the *tnp* gene is little expressed, limiting ISPpu9 activity. Certainly, *tnp* mRNA showed a short half-life (∼3 min) and was present at very low levels (Figure 2A). Low translational output derived from a *tnp* 5′-UTR secondary structure that hinders translation initiation might accelerate degradation of *tnp* mRNA by RNases.

Finally, ISPpu9 appeared to be organized as two adjacent segments flanked by the highly conserved ‘A’ and ‘B’ boxes described in Figure 1; one segment includes *asr9* and *tnp*, and the other *ssr9*. Since strains KT2440, KBS0802 and NCTC13186 have ISPpu9 copies with or without *ssr9*, and *ssr9* can be present at other sites not necessarily associated with the *tnp* gene, it is tempting to suggest that ISPpu9 might mobilize as an A-B segment made up of either three elements (*tnp*, *asr9* and *ssr9*), two elements (*tnp* and *asr9*) or just one (*ssr9*). However, it remains unclear whether the independent *ssr9* copies present arose by mobilization of a pre-existing *ssr9*-containing ISPpu9 copy that left behind the *ssr9* gene, or by an independent mobilization of the *ssr9* A-B segment helped by a *tnp* gene.

## DATA AVAILABILITY

The genome sequences analyzed are available at https://www.pseudomonas.com (54).

The ARNold software (52) used to predict the stem-loop structures that might act as Rho-independent transcriptional terminators is available at http://rssf.i2bc.paris-saclay.fr/toolbox/arnold/.

The RNAFold software (78) used to predict RNA secondary structure is available at http://rna.tbi.univie.ac.at/cgi-bin/RNAWebSuite/RNAfold.cgi.

The intaRNA software (79) used to identify regions of sequence complementarity between the sRNAs is available at http://rna.informatik.uni-freiburg.de.

## Supplementary Material

gkab672_Supplemental_File
