# Supplementary material for: Expression of the ISPpu9 transposase of Pseudomonas putida KT2440 is regulated by two small RNAs and the secondary structure of the mRNA 5′-untranslated region
Source: Nucleic Acids Res. 2021 Aug 11;49(16):9211–28. doi: 10.1093/nar/gkab672 (PMC8450116; doi:10.1093/nar/gkab672)
Supplement: gkab672_Supplemental_File [file gkab672_supplemental_file.pdf]

Supplementary material for:

Expression of the ISPpu9 transposase of *Pseudomonas putida* KT2440 is regulated by two small RNAs and the secondary structure of the mRNA 5´-untranslated region

Guillermo Gómez-García[1], Angel Ruiz-Enamorado[1], Luis Yuste[1], Fernando Rojo[1,*] and Renata Moreno[1,*]

[1] Department of Microbial Biotechnology, Centro Nacional de Biotecnología, CSIC, Madrid - 28049, Spain

* To whom correspondence should be addressed. Tel: +34 91 585 4539; Fax: +34 91 585 4506; Email: frojo@cnb.csic.es. Correspondence may also be addressed to R. Moreno. Tel: +34 91 585 4571; Fax: +34 91 585 4506; Email: rmoreno@cnb.csic.es

Table S1. Oligonucleotides used

Oligonucleotide sequence (5´–3´)	Name	Use
**Probes for Northern assays**		
gaaag*aagctt*tagaccgcactggtgtg	ssr9A-D	RNA probe Northern Ssr9
ggggt*gaattc*cggcgaagtgttcactc	ssr9A-R	RNA probe Northern Ssr9
tta*gaattc*gtgtgcgcgggac	asr9A-D	RNA probe Northern Asr9
ctcg*aagctt*aaagggcgcgcg	asr9A-R	RNA probe Northern Asr9
**S1 nuclease protection assays**		
gattgtctgtgcattgctgct	tnp1-D	S1 protection assay for *tnp*; direct primer
atcagctgatgcctacaggga	tnp1-R	S1 protection assay for *tnp*; reverse primer
aggagttcgaaaccagaagcc	ssr9B-D	S1 protection assay for *ssr9*; direct primer
tttgggccactctctcattcg	ssr9B-R	S1 protection assay for *ssr9*; reverse primer
agccagttagccatcttgagc	asr9B-D	S1 protection assay for *asr9*; direct primer
acagagatcgtagccagcact	asr9B-R	S1 protection assay for *asr9*; reverse primer
**Fusions to *lacZ* or *'lacZ***		
gta*ggatcc*cacccagcccttcc	cmFus-D	Transcriptional fusion *Ptnp-lacZ*, and translational fusions *tnp'-'lacZ*, direct primer
tgatg*aagctt*cgcaggataaag	Ptnp-R	Transcriptional fusion *Ptnp-lacZ*, reverse primer
ttgctct*ggatcc*gacgagataaac	Pasr9-D	Transcriptional fusion *Pasr9-lacZ*, direct primer
cgtat*aagctt*gtgaaccctttcgc	Pasr9-R	Transcriptional fusion *Pasr9-lacZ*, reverse primer
gtgctta*ggatcc*aatgggtt	Pssr9-D	Transcriptional fusion *Pssr9-lacZ,* direct primer
gcggtctacggc*aagctt*ccc	Pssr9-R	Transcriptional fusion *Pssr9-lacZ*, reverse primer
tggaa*aagctt*cgcgccatgat	2C-R	*tnp'-'lacZ* translational fusion in pTNL-tnp2, reverse primer
cggta*aagctt*tgcttggaaggcttg	8C-R	*tnp'-'lacZ* translational fusion in pTNL-tnp8, reverse primer
cggtaaagctttgtttactcggcttg	8C-35-R	*tnp'-'lacZ* translational fusion in pTNL-tnp8m35, reverse primer
**RT-qPCR assays**		
agctgatgcctacagggacttc	qPCR-asr9-D	RT-qPCR for *asr9*, direct primer
tgcacacccgcgtagga	qPCR-asr9-R	RT-qPCR for *asr9*, reverse primer
tccctgtcggcacagctt	qPCR-ssr9-D	RT-qPCR for *ssr9*, direct primer
ggcggcgaagtgttcact	qPCR-ssr9-R	RT-qPCR for *ssr9*, reverse primer
atcaggatatgtggcggatga	qPCR-lacZ-D	RT-qPCR for *lacZ*, direct primer
tgatttgtgtagtcggtttatgca	qPCR-lacZ-R	RT-qPCR for *lacZ*, reverse primer
tcgacccggagctggata	rpoN-fwd	RT-qPCR *rpoN*
cggctcgaactgctggat	rpoN-rev	RT-qPCR *rpoN*
gctgcgtccggtcattg	tnp-fwd	RT-qPCR *tnp*
gcgcctggtctttcacttg	tnp-rev	RT-qPCR *tnp*
ttgacgaccatagagcattgga	5S-fwd	RT-qPCR 5s rRNA
gatgcatcgtttcactactgagtt	5S-rev	RT-qPCR 5s rRNA
**Production of *asr9* and *ssr9 by in vitro* transcription**		
tagggcaagcttccttttgctttgttagg	asr9-D	Amplification of *asr9* for pSPT-AS, direct primer
ctcgcagaattcagggcgcgcgg	asr9-R	Amplification of *asr9* for pSPT-AS, reverse primer

ggttccgaattcgcgggaaagag	ssr9-D	Amplification of *ssr9* for pSPT-S, direct primer
aaagataagcttctgttctttctagcgaa	ssr9-R	Amplification of *ssr9* for pSPT-S, reverse primer
**Amplification of *ssr9* and *asr9***		
tagaaaactagtggttgcattttgtttgatga	ssr9C-D	*ssr9* as a SpeI fragment to obtain pGEMTssr9; direct primer
ccgtttactagtgcctttgccggccctt	ssr9C-R	*ssr9* as a SpeI fragment to obtain pGEMTssr9; reverse primer
gctcctactagtacggcgcagaat	asr9C-D	*asr9* as a SpeI fragment to obtain pGEMTasr9; direct primer
gggatggactagtgtaaagcgtt	asr9C-R	*asr9* as a SpeI fragment to obtain pGEMTasr9; reverse primer
**Construction of ISPpu9-Km and its derivatives**		
a*gaattc*gaaatctgtggggg	ISPpu9-D	Construction of pKN-ISPpu9Km and its derivatives; direct primer
gaccgtttgatggtgcctttg	ISPpu9-R	Construction of pKN-ISPpu9Km and its derivatives; reverse primer
ggcgtttttattgtctagaatcc	Km-D	Kanamycin amplification (transposition assays)
aagcagggttctagagcggaaaag	Km-R	Kanamycin amplification (transposition assays)
cgccgaataagagtgaacacttcgccg	ISPpu9ΔS-D	Construction of pKN-ISPpu9KmΔ*ssr9*, direct primer, short fragment
gttcactcttattcggcggctgaccta	Δssr-R	Construction of pKN-ISPpu9KmΔ*ssr9*; 3'-end reverse primer
ctgtttgctggtaaagcgttgcttggaa	Δtnp-R	Construction of pKN-ISPpu9KmΔ*tnp*; 3'-end reverse primer
acgctttaccagcaaacaggatgcgca	Δtnp-D	Construction of pKN-ISPpu9KmΔ*tnp*; 3'-end direct primer

A

Strain	*tnp*	*asr9*	*ssr9*
KT2440	7	7	8
KBS0802	7	7	9
NCTC13186	7	7	12

B

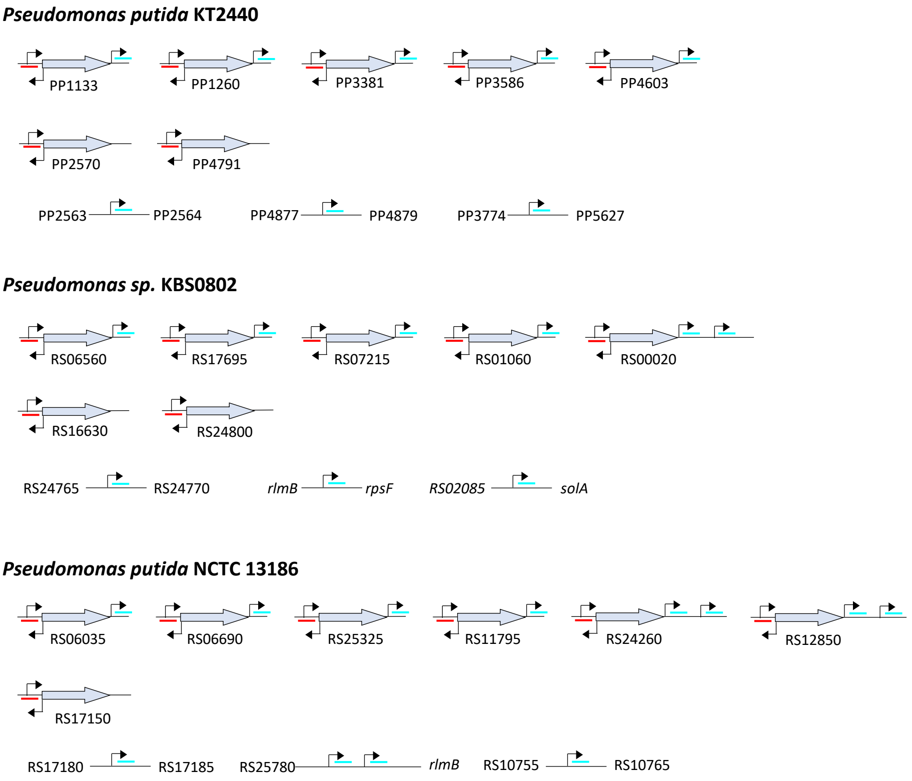

Figure S1. Number (**A**) and distribution (**B**) of ISPpu9 or ISPpu9-like sequences (*tnp*, *asr9*, *ssr9*) in strains *P. putida* KT2440, *Pseudomonas sp*. KBS0802 and *P. putida* NCTC13186. ISPpu-like sequences for strains KSB802 and NCTC13186 were deduced by sequence similarity to strain KT2440 ISPpu9. All ISPpu9-like sequences shown for strains KBS0802 and NCTC13186 had *tnp* and *asr9* genes with 100% nucleotide identity to those of PP_3381, which is the strain KT2440 ISPpu9 copy used as query in the searches made. In the case of *ssr9*, all copies presented were 100% identical to those of PP_3381 with two exceptions: the *ssr9* copy of strain KSB0802 located downstream of RS02085, and that of strain NCTC13186 located downstream of RS10755, which were 98.4 identical to the ssr9 gene present in PP_3381.

The transposase gene (*tnp*) is indicated with grey arrows, *asr9* is in red and *ssr9* in blue. Thin arrowheads indicate the promoters identified in KT2440; their location in strains KBS0802 and NCTC13186 is only tentative and based on the experimental results for strain KT2440.
